# Comparative Immune Response after Vaccination with SOBERANA^®^ 02 and SOBERANA^®^ plus Heterologous Scheme and Natural Infection in Young Children

**DOI:** 10.3390/vaccines11111636

**Published:** 2023-10-25

**Authors:** Rocmira Pérez-Nicado, Chiara Massa, Laura Marta Rodríguez-Noda, Anja Müller, Rinaldo Puga-Gómez, Yariset Ricardo-Delgado, Beatriz Paredes-Moreno, Meiby Rodríguez-González, Marylé García-Ferrer, Ilianet Palmero-Álvarez, Aniurka Garcés-Hechavarría, Daniel G. Rivera, Yury Valdés-Balbín, Vicente Vérez-Bencomo, Dagmar García-Rivera, Barbara Seliger

**Affiliations:** 1 Finlay Vaccine Institute, 200 and 21 Street, Havana 11600, Cuba; rpnicado@finlay.edu.cu (R.P.-N.); lmrodriguez@finlay.edu.cu (L.M.R.-N.); bparedes@finlay.edu.cu (B.P.-M.); mcrodriguez@finlay.edu.cu (M.R.-G.); mgarcia@finlay.edu.cu (M.G.-F.); ipalmero@finlay.edu.cu (I.P.-Á.); agarces@finlay.edu.cu (A.G.-H.); yvbalbin@finlay.edu.cu (Y.V.-B.); vicente.verez@finlay.edu.cu (V.V.-B.); 2 Institute for Translational Immunology, Brandenburg Medical School “Theodor Fontane”, 14770 Brandenburg, Germany; chiara.massa@medizin.uni-halle.de; 3 Medical Faculty, Martin Luther University, 06112 Halle (Saale), Germany; anja.mueller@uk-halle.de; 4 Pediatric Hospital “Juan Manuel Márquez”, Havana 11500, Cuba; puga@infomed.sld.cu (R.P.-G.); yariricardo@infomed.sld.cu (Y.R.-D.); 5 Laboratory of Synthetic and Biomolecular Chemistry, Faculty of Chemistry, University of Havana, Havana 10400, Cuba; danielgr@fq.uh.cu; 6 Fraunhofer Institute for Cell Therapy and Immunology, 04103 Leipzig, Germany

**Keywords:** SARS-CoV-2, COVID-19, vaccine, children, SARS-CoV-2 RBD IgG, memory T cell

## Abstract

(1) Background: In children, SARS-CoV-2 infection is mostly accompanied by mild COVID-19 symptoms. However, multisystem inflammatory syndrome (MIS-C) and long-term sequelae are often severe complications. Therefore, the protection of the pediatric population against SARS-CoV-2 with effective vaccines is particularly important. Here, we compare the humoral and cellular immune responses elicited in children (*n* = 15, aged 5–11 years) vaccinated with the RBD-based vaccines SOBERANA^®^ 02 and SOBERANA^®^ Plus combined in a heterologous scheme with those from children (*n* = 10, aged 4–11 years) who recovered from mild symptomatic COVID-19. (2) Methods: Blood samples were taken 14 days after the last dose for vaccinated children and 45–60 days after the infection diagnosis for COVID-19 recovered children. Anti-RBD IgG and ACE2-RBD inhibition were assessed by ELISA; IgA, cytokines, and cytotoxic-related proteins were determined by multiplex assays. Total B and T cell subpopulations and IFN-γ release were measured by multiparametric flow cytometry using a large panel of antibodies after in vitro stimulation with S1 peptides. (3) Results: Significant higher levels of specific anti-RBD IgG and IgA and ACE2-RBD inhibition capacity were found in vaccinated children in comparison to COVID-19 recovered children. Th1-like and Th2-like CD4^+^ T cells were also significantly higher in vaccinated subjects. IFN-γ secretion was higher in central memory CD4^+^ T cells of COVID-19 recovered children, but no differences between both groups were found in the CD4^+^ and CD8^+^ T cell effector, terminal effector, and naïve T cell subpopulations. In contrast to low levels of IL-4, high levels of IL-2, IL-6, IFN-γ, and IL-10 suggest a predominant Th1 cell polarization. Cytotoxic-related proteins granzyme A and B, perforin, and granulin were also found in the supernatant after S1 stimulation in both vaccinated and recovered children. (4) Conclusions: Vaccination with the heterologous scheme of SOBERANA^®^ 02/SOBERANA^®^ Plus induces a stronger antibody and cellular immune response compared to natural infections in young children.

## 1. Introduction

The COVID-19 pandemic caused by the severe acute respiratory syndrome coronavirus 2 (SARS-CoV-2) was a global emergency, with more than 6 million deaths [1]. The pediatric population develops mostly mild or moderate symptoms in SARS-CoV-2 infections, but there is evidence of severe complications such as multisystem inflammatory syndrome (MIS-C) and long-term sequelae (“long COVID”) [2,3,4]. Children also play an important role in the transmission of the disease to other vulnerable groups, such as people over 65 years [5,6].

With more than 13 billion doses administrated so far, vaccination against COVID-19 in the adult population has been extended worldwide, but only 32.6% of the population in low-income countries has received at least one vaccine dose [7]. Several vaccines have been approved for children by local and global agencies; mRNA-based vaccines and virus-inactivated vaccines have been the most applied in the pediatric population over 5 years old [8,9,10]. Due to their safety and efficacy, vaccines based on protein subunits are very attractive for childhood vaccination, as demonstrated through the millions of doses administered against typical childhood infectious diseases like pneumonia or meningitis [11]. SOBERANA^®^ 02 (based on the SARS-CoV-2 recombinant receptor-binding domain (RBD) chemically conjugated to tetanus toxoid [12]) and SOBERANA^®^ Plus (based on the recombinant RBD-dimer [13]) are two protein subunit vaccines against SARS-CoV-2 produced by the Finlay Institute of Vaccines in Havana, Cuba. The heterologous combination of two doses of SOBERANA^®^ 02 followed by one dose of SOBERANA^®^ Plus 28 days apart is safe, immunogenic, and effective in adults 19–80 y/o [14,15,16] and in children 3–18 y/o [17]. This scheme prevented COVID-19 disease in adults with an efficacy of 92% protection against symptomatic disease [14].

A phase I/II clinical trial in 3–18 y/o children evaluated the humoral immune response as well as the cytokine secretion induced by this heterologous scheme [17]. Here, we characterize the humoral and cellular responses by measuring antigen-specific B and T cell subpopulations in a subset of children vaccinated with the heterologous scheme during the clinical trial, and we compare these responses with those in children recovered from mild symptomatic COVID-19. This study introduces novel aspects of this RBD-based vaccine combination in children, including specific IgA concentration, a titer of neutralizing antibodies against the variants of concern (VOCs) Delta and Omicron BA.1, and frequencies of specific memory B and effector CD4^+^ and CD8^+^ T cells.

## 2. Materials and Methods

### 2.1. Subjects and Ethics

A subgroup of children between 5 and 11 years (*n* = 15) were randomly selected from participants in a phase I/II clinical trial conducted at the “Juan Manuel Marquez” Pediatric Hospital in Havana, Cuba [17] (trial registry: https://rpcec.sld.cu/trials/RPCEC00000374-En, accessed on 9 August 2023). They were vaccinated every 28 days with the heterologous scheme of two doses of SOBERANA^®^ 02 and a third dose with SOBERANA^®^ Plus. The study was conducted on October 2021 during the prevalence of the Delta variant and before the appearance of the Omicron variant [18]. Key inclusion criteria were weight, height, nutritional assessment, physical examination without alterations, clinical laboratory results within the range of reference values, and microbiology laboratory tests. The key exclusion criteria were any acute infection, a previous or current history of SARS-CoV-2 infection, and being in contact with a positive COVID-19 case. Reverse transcriptase-polymerase chain reaction (PCR) for SARS-CoV-2 detection was performed in all participants at least 72 h before each dose. A detailed description of the selection criteria has been described previously [17].

Additionally, as a control for the immunity caused by SARS-CoV-2 natural infection, 10 children aged 4–11 y/o, who had suffered from mild symptomatic COVID-19 confirmed by RT-PCR 45–60 days before blood draw, were recruited in a follow-up medical consultation for convalescent children at the hospital where the clinical trial was conducted. They were not participants in the clinical trial. They all were infected during the predominance of the Delta variant. These subjects reported a single previous infection confirmed by RT-PCR to SARS-CoV-2.

Their parents signed an informed consent to include their children in this study and the results were communicated to them. The medical investigators provided the parents with all the information about the vaccination and its potential risks and benefits.

### 2.2. Blood Collection and Isolation of Peripheral Blood Mononuclear Cells

Blood samples were taken 14 days after the last dose in vaccinated children, and 45–60 days after the infection diagnosis (by RT-PCR for SARS-CoV-2) for the control group (children who had recovered from symptomatic mild COVID-19).

Blood samples were collected by venipuncture in a EDTA-K_3_ coated tubes Vacutainer™ (Greiner Bio-One, Kremsmünster, Austria). Peripheral blood mononuclear cells (PBMCs) were isolated by differential centrifugation in a gradient of Ficoll-Paque™ Plus (GE-Healthcare, Uppsala, Sweden) following the manufacturer’s guidelines. Briefly, anti-coagulated PBMCs were carefully layered over Ficoll and centrifuged at 400× *g* during 20 min at room temperature. The plasma fraction was removed for antibody determinations and stored at −80 °C until use. The PBMC-rich layer was carefully removed, washed multiple times, centrifuged to remove platelets, and then stored in a cryopreservation medium containing heat-inactivated fetal bovine serum (FBS, Gibco, Bleiswijk, The Netherlands) and 10% DMSO (Sigma-Aldrich, St. Louis, MI, USA) in liquid nitrogen until use.

### 2.3. Antibody Determinations

Plasma samples were tested for an S-specific IgG response using the quantitative electrochemiluminescence immunoassay Elecsys^®^ Anti SARS-CoV-2 S test and the Cobas e411 Analyzer (Roche Diagnostics, Mannhein, Germany) following the manufacturer’s guidelines. The manufacturer specific U/mL of the Elecsys^®^ Anti-SARS-CoV-2 S assay can be considered equivalent to the Binding Arbitrary Units (BAU/mL) of the first WHO International Standard for anti-SARS-CoV-2 immunoglobulin (NIBSC 20/136). The antibodies’ capacity for blocking the RBD-hACE2 interaction was assessed with a competitive ELISA [17]. RBD-specific IgA was determined in plasma using the bead-based multiplex assay, LEGENDplex SARS-CoV-2 Serological IgA Panel (2-plex) Spike (S1) (Biolegend™, San Diego, CA, USA) following the manufacturer’s instructions. Except for the conventional viral neutralization assays, proteins with the Wuhan variant sequence were used. The conventional virus neutralization test was assessed in a biosecurity laboratory level 3 (National Civil Defense Research Laboratory, Havana, Cuba) [17].

### 2.4. Staining of Cells and Multicolor Flow Cytometry

PBMCs were characterized by multiparametric flow cytometry for B and T cell subpopulations. The FITC anti-human CD19 (HIB19, BD Pharmingen™, San Diego, CA, USA), PE-Cy7 anti-human IgD (IA6-2, BioLegend™, San Diego, CA, USA), BV421 anti-human CD27 (O323, BioLegend™), V500 anti-human CD45 (HI30, BD Horizon™, San Diego, CA, USA), and BV605 anti-human CD3 (OKT3, BioLegend™) were used to identify total B cell populations. T helper polarization markers were assessed with an FITC anti-human CD3 (SK7, BD Biosciences, San Diego, CA, USA), PE anti-human CD183 (CXCR3) (1C6/CXCR3, BD Pharmingen™), PE-Cy7 anti-human CD196 (CCR6) (G034E3, BioLegend™), APC anti-human CD4 (RPA-T4, BD Pharmingen™), APC-H7 anti-human CD8 (SK1, BD Pharmingen™), V500 anti-human CD45 (HI30, BD Horizon™), and BV605 anti-human CD194 (CCR4) (1G1, BD Biosciences) antibody cocktail. Representative gating strategies are shown in Figures S1 and S2. No live/dead exclusion gate was present because trypan blue staining was used for the exclusion of dead cells. The percentage of dead cells was lower than 10%.

Specific B cell analysis was assessed with double tetramer staining using an RBD-specific B cell kit (Miltenyi-Biotec, Bergisch Gladbach, Germany, 130-128-032), following the manufacturer’s instructions. Data were acquired on a MACSQuant16 Flow Cytometer (Miltenyi-Biotec) and analyzed by FlowJo software X 10.0.7 (FlowJo LLC, Ashland, OR, USA). Live singlets CD19^+^ lymphocytes were gated based on 7AAD fluorescence and specific B cells. Cells incubated with streptavidin PE and PEVio770 alone were used as negative controls. IgG^+^, IgM^+^, and IgA^+^ specific memory B cells were defined as CD27^+^ CD19^+^ on tetramer^+^ B cells.

### 2.5. IFN-γ Secretion Assay for an Evaluation of the T Cell Specific Response 

PBMCs were thawed and their viability was determined after trypan blue staining in a hemocytometer. Cells were adjusted to 1 × 10^7^ cells/mL in X-Vivo 15 (Lonza, Basel, Switzerland) supplemented with 1 mM of sodium pyruvate (Sigma-Aldrich), 1 mM of L-glutamine (Sigma-Aldrich), and 1% of penicillin/streptomycin (Sigma-Aldrich), which is then either left untreated or stimulated with 1 µg/mL of a SARS-CoV-2 S1 peptide pool (PepTivator^®^ SARS-CoV-2 Spike Protein Peptide Pool Miltenyi-Biotec) consisting mainly of 15-mer sequences with 11 amino acids overlap, covering the immunodominant sequence domains of the spike glycoprotein (“S1”) of SARS-CoV-2) or left untreated. After 16–18 h at 37 °C in 5% CO_2_, the culture supernatants were collected and stored at −80 °C, whereas the cells were evaluated with an IFN-γ secretion kit assay (Miltenyi-Biotec) designed for the detection and analysis of viable IFN-γ-secreting leukocytes, following the manufacturer’s recommendations. Briefly, the cells were stained for 5 min on ice with the anti-human IFN-γ capture antibody and incubated for 45 min at 37 °C under slow continuous rotation followed by washing and staining with anti-human IFN-γ-APC (Miltenyi-Biotec) for 10 min on ice. In order to dissect the cell populations secreting IFN-γ and their activation and memory status, the cells were also stained with PE anti-human CCR7 (150503, BD Pharmingen™), PerCP/Cy5.5 anti-human CD4 (RPA-T4, BioLegend™), PE-Cy 7 anti-human CD45RA (HI100, BD Pharmingen™), APC-H7 anti-human CD8 (SK1, BD Pharmingen™), V500 anti-human CD45 (HI30, BD Horizon™), and BV605 anti-human CD3 (OKT3, BioLegend™) antibodies. The cells were then acquired on an LSR Fortessa Flow Cytometer (BD Biosciences) and the data were analyzed with FACS Diva 8.0 and FlowJo X 10.0.7 software (BD Biosciences). Representative gating strategies are shown in Figure S3.

### 2.6. Determination of Cytokine Release

For the determination of the released cytokines, the supernatants of the cell cultures stimulated with PepTivator Peptide Pools were analyzed using a LEGENDplex CD8/NK cytokine-profile 13-plex kit (Biolegend) according to the manufacturer’s instructions. Cytokine levels were normalized by removing the values from unstimulated controls. Data were analyzed with the LEGENDplex v8.0 Software (Biolegend). The method has been described in detail [19].

### 2.7. Statistics

The statistical analysis was performed using GraphPad Prism 9.0 (GraphPad Software, San Diego, CA, USA). Data were described using geometric means and medians with 95% confidence intervals. Non-paired samples were analyzed with a non-parametric Mann–Whitney test.

## 3. Results

### 3.1. Characterization of the Study Cohort

**Figure 1:**
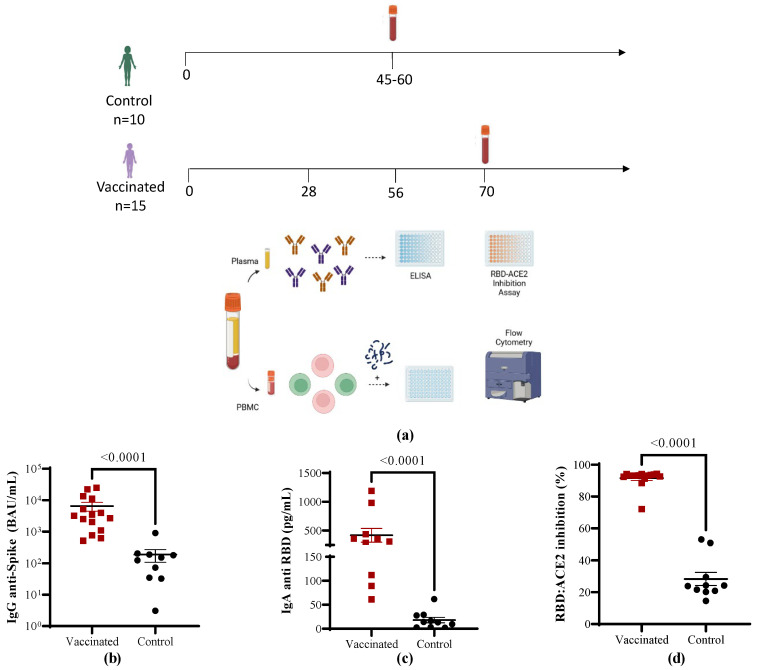
Spike specific antibody response in vaccinated and COVID-19 recovered children. (**a**) Experimental design. Blood samples were collected from vaccinated children (*n* = 15) 14 days after the last (third) dose of the SOBERANA^®^ 02/SOBERANA^®^ Plus heterologous scheme, and from children (control, *n* = 10) who recovered from mild COVID-19 (45–60 days after the infection diagnosis). (**b**) Spike IgG antibodies expressed in BAU/mL. (**c**) Concentration of anti-RBD IgA antibodies in pg/mL. (**d**) Serum-induced inhibition of the RBD–hACE2 interaction. Mean ± SEM are shown together with the *p*-values from the Mann–Whitney non-parametric *t* test.

Children aged 4–11 years old who recovered from COVID-19 (*n* = 10) or were vaccinated with the heterologous scheme SOBERANA^®^ 02/SOBERANA^®^ Plus (*n* = 15) were recruited in order to analyze in depth their humoral and cellular immune responses. Their demographic characteristics are presented in Table 1. The study design is summarized in Figure 1a.

### 3.2. Antibody Immune Responses in Vaccinated Children Compared to COVID-19 Recovered Children 

To characterize the antibody response, anti-spike IgG levels were determined with an electrochemiluminescence assay calibrated with an international reference serum. The children vaccinated with SOBERANA^®^ 02/SOBERANA^®^ Plus had higher IgG antibody titers against RBD compared to COVID-19 convalescent children (Figure 1b, *p* < 0.0001). In line with the anti-RBD IgG response, the levels of anti-RBD IgA were also higher in the vaccinated group than after natural infection (Figure 1c, *p* < 0.0001).

**Figure 2:**
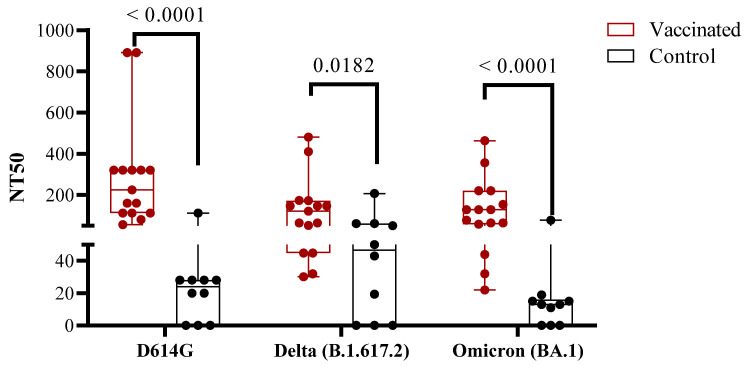
Neutralizing antibody response in vaccinated and COVID-19 recovered children. Mean ± SEM are shown together with the *p*-values from the Mann–Whitney non-parametric *t* test.

We evaluated the neutralizing capacity of antibodies for blocking the binding of the RBD to its receptor using a competitive immunoassay, which measures the percentage of the inhibition of RBD–hACE2 interaction. As shown in Figure 1d, all the vaccinated individuals performed better than the children who recovered from the disease (*p* < 0.0001). The neutralizing titer against the variants D614G, Delta, and Omicron (BA.1) was also evaluated by the conventional neutralization assay. Concerning the inhibition capacity of the antibodies, the neutralizing titer in vaccinated children was higher than in the COVID-19 recovered children (against D614G, *p* < 0.0001, against Delta, *p* = 0.0182, and against Omicron (BA.1), *p* < 0.0001) (Figure 2).

### 3.3. Total and RBD-Specific B Cells and T Helper Populations in Vaccinated and COVID-19 Recovered Children

In order to characterize the functional memory B cell response, we first analyze the frequency of the total B cell subpopulations on PBMCs from vaccinated and COVID recovered children using multiparametric flow cytometry. The gating strategy is shown in Figures S1 and S2. No significant differences were found between vaccinated and recovered children in the frequency of total naïve (IgD^+^CD27^−^), exhausted (IgD^−^CD27^−^), and pre-switched B cells (IgD^+^CD27^+^), or switched memory plasmablast (CD24^−^CD38^+^) (Figure S4A–E) and transitional naïve B cells (CD24^+^CD38^+^) (Figure S4F), but significant higher levels (*p* = 0.0044) of switched memory B cells (IgD^−^CD27^+^) were found in vaccinated children (Figure S4D).

**Figure 3:**
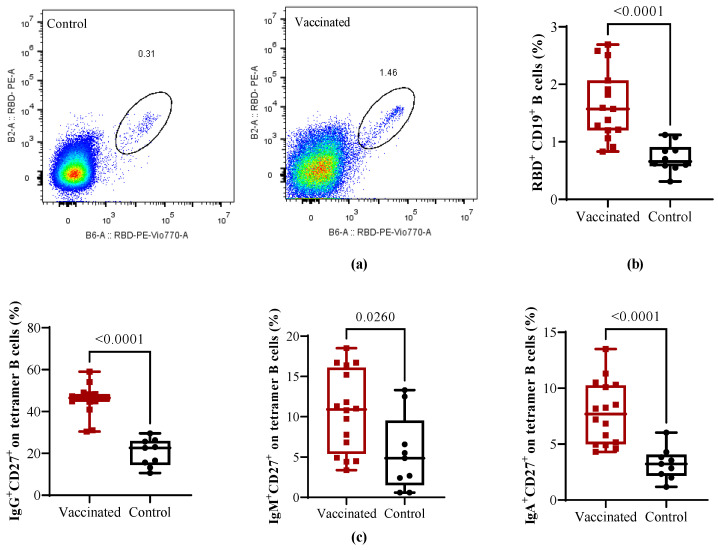
Analysis of B cell frequency in vaccinated and COVID-19 recovered children. (**a**) Representative gate of RBD-specific B cells in recovered and vaccinated children. (**b**) Percentages of RBD tetramer^+^ on CD19^+^ B cells. (**c**) Percentages of IgG^+^CD27^+^, IgM^+^CD27^+^, and IgA^+^CD27^+^ on RBD^+^ B cells. Mean ± SEM are shown together with the *p*-values from the Mann–Whitney non-parametric *t* test.

RBD-specific memory B cells were analyzed by flow cytometry with a double tetramer staining (Figure 3a as representative). Compared to recovered children, in vaccinated individuals, the total RBD-specific circulating CD19^+^ B cells were significantly higher (*p* < 0.0001), as seen in Figure 3b. Further analyses of the specific CD27^+^ memory B cell subsets show that in comparison with naturally infected controls, the frequencies of isotype memory subpopulations IgG^+^CD27^+^ (*p* < 0.0001), IgM^+^CD27^+^ (*p* < 0.0260), and IgA^+^CD27^+^ B cells (*p* < 0.0001) meant that cells were significantly higher in vaccinated children, as shown in Figure 3c.

**Figure 4:**
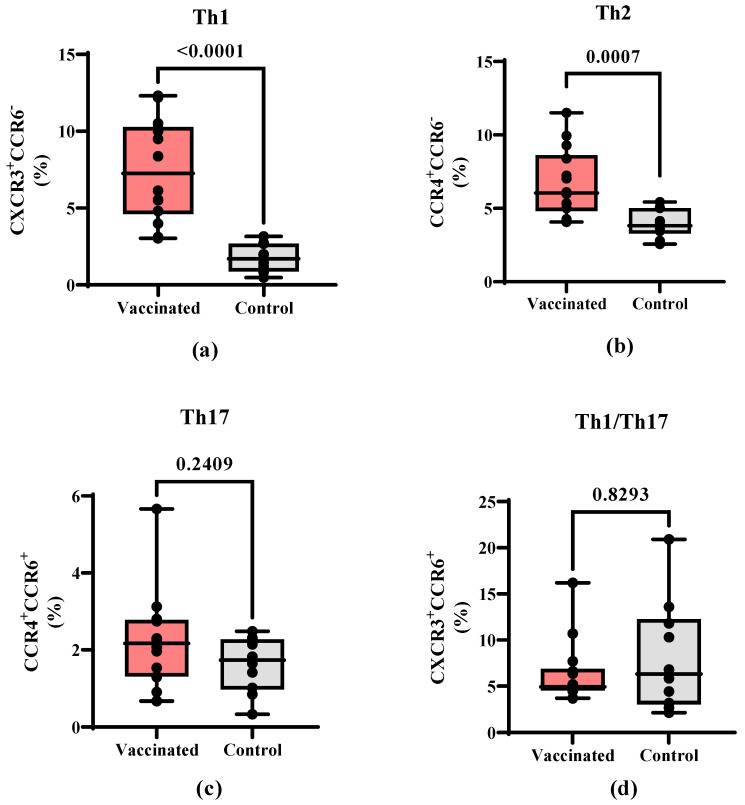
Composition of the T helper cell subpopulation in vaccinated and COVID-19 recovered children. PBMCs from vaccinated and recovered children (control) were isolated and stained with antibody cocktails for CD4^+^ T cell subpopulations. The frequencies of Th1 CD4^+^ T cells (CD4^+^CXCR3^+^CCR6^−^) are shown. (**a**) Th2 CD4^+^ T cells (CD4^+^CCR4^+^CCR6^−^), (**b**) Th17 CD4^+^ T cells (CD4^+^CCR4^+^CCR6^+^), (**c**) Th1/Th17 cells (CD4^+^CXCR3^+^CCR6^+^), and (**d**) CD4^+^ T cells for individual children. The data are also shown as mean ± SEM and the *p*-values from the Mann–Whitney non-parametric *t* test.

In addition, CD4^+^ T cell subpopulations were further characterized to compare the frequencies of the different T helper cell polarization profiles. Th1-like (CD4^+^CXCR3^+^CCR6^−^, *p* < 0.0001) cells (Figure 4a) and Th2-like (CD4^+^CCR4^+^CCR6^−^, *p* = 0.0007) cells (Figure 4b) showed a higher percentage of cells in vaccinated children. In contrast, no statistically significant differences were found in the Th17-like (CD4^+^CCR4^+^CCR6^+^) (Figure 4c) and Th1/Th17-like subpopulations (CD4^+^CXCR3^+^CCR6^+^) between both groups (Figure 4d).

### 3.4. Functional Properties of T Cells after Antigen-Specific In Vitro Stimulation

**Figure 5:**
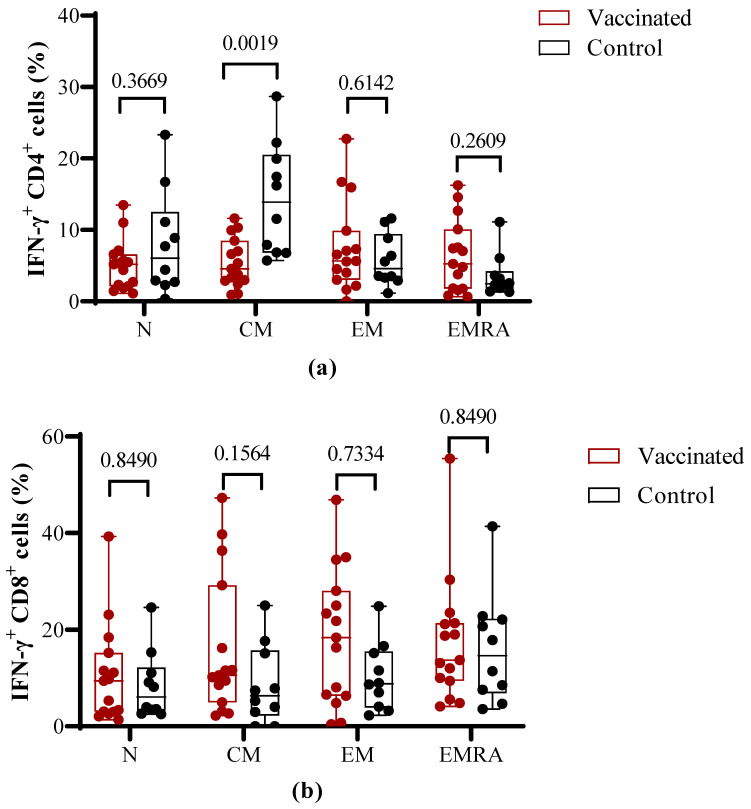
Percentage of IFN-γ secreting CD4^+^ and CD8^+^ memory T cells in vaccinated and COVID-19 recovered children. PBMCs from convalescent and vaccinated children were left unstimulated or were stimulated with S1 SARS-CoV-2 peptide pool, then the T cell-specific response was evaluated as secretion of IFN-γ within the CD4^+^ (**a**) and CD8^+^ (**b**) T cell subpopulations with respect to their memory phenotype. The bars show the mean ± SEM values and the *p*-values from the Mann–Whitney non-parametric *t* test. N: naive; CM: central memory; EM: effector memory; EMRA: terminal differentiated effector memory.

To compare the effector properties of T cells induced by vaccination versus natural infection, PBMCs were stimulated in vitro with the S1 peptide pool and then evaluated by flow cytometry for IFN-γ release and the expression of T cell memory markers. Within the CD4^+^ T cell populations, no statistical differences on IFN-γ release were found for naïve effector memory (EM) and terminally differentiated effector memory (EMRA) between vaccinated and COVID-19 recovered children. In contrast, in the central memory (CM) CD4^+^ T cell subpopulation, a higher IFN-γ secretion (*p* = 0.0019) was detected in COVID-19 recovered children (Figure 5a).

The percentage of activated IFN-γ secreting CD8^+^ central memory (CM) and effector memory (EM) T cells did not show a statistically significant difference (*p* > 0.05) between vaccinated and COVID-19 recovered children. Nevertheless, the relative percent is higher for CD8^+^ (CM) and CD8^+^ (EM) in the vaccinated individuals in comparison to recovered children. The percentage of IFN-γ secreting CD8^+^ (EMRA) and CD8^+^ (naïve) cells did not show statistical differences with controls (Figure 5b).

**Figure 6:**
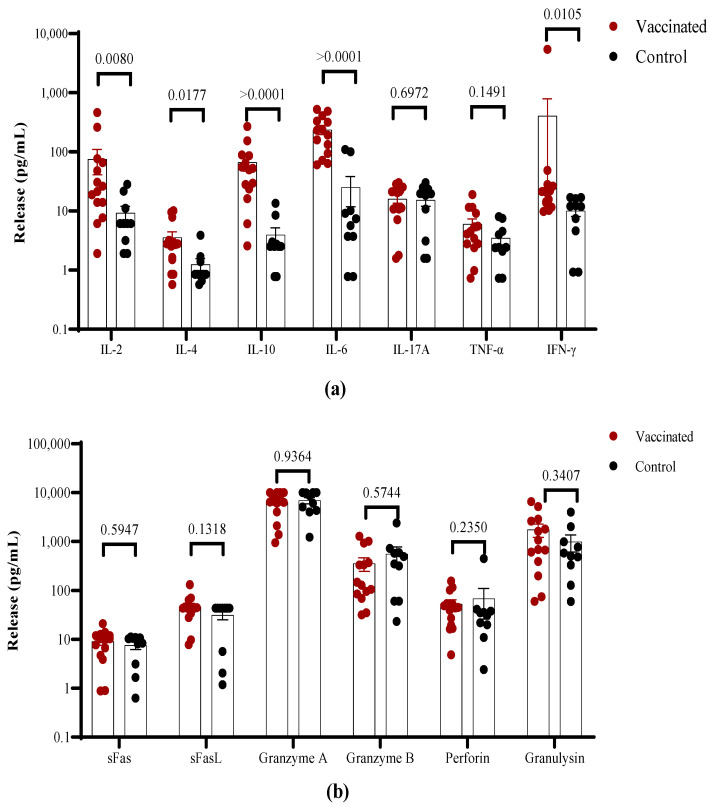
Spike-specific cytokine secretion in vitro from vaccinated and COVID-19 recovered children. PBMCs from convalescent and vaccinated children were stimulated with a S1 SARS-CoV-2 peptide pool. Cell supernatants were analyzed using a multiplex cytokine secretion assay. (**a**) Cytokines; (**b**) cytotoxicity-related molecules. Each dot identifies an individual child; the bars indicate mean ± SEM and *p*-values from the Mann–Whitney non-parametric *t* test.

The overall cytokine profile produced in response to stimulation with the S1 peptide pool was analyzed in the T cell culture supernatants using a multiplex assay. Higher levels of IFN-γ, IL-2, IL-10, and IL-6, but not TNF-α, were found in vaccinated children in comparison to recovered individuals (Figure 6a). The higher IFN-γ levels in vaccinated children are consistent with the higher frequency of responding cells. In general, low levels of IL-4 were found in both groups with values <10 pg/mL. No differences were detected in IL-17A (Figure 5a). Furthermore, higher levels of the cytotoxicity-associated molecules perforin, sFas/sFasL, granulysin, as well as granzyme A and B were detected in both groups (Figure 6b).

## 4. Discussion

After three years of the COVID-19 pandemic, vaccination against SARS-CoV-2 in pediatric populations has not been extended worldwide. Two doses of SOBERANA^®^ 02 in the heterologous scheme with a third dose of SOBERANA^®^ Plus has shown an excellent safety profile and immunogenicity in children aged 3–18 years [17]. This heterologous vaccination scheme is characterized by high levels of anti-RBD antibodies with neutralizing capacity against VOCs (including BA.1) and IFN-γ secreting T cells [17]. These results supported the emergency use authorization and their use in a massive vaccination campaign covering more than 96% of the pediatric population aged 2–18 years in Cuba [17,20].

The novelty of this study lies in the comprehensive characterization of SARS-CoV-2 specific humoral and cellular immune responses in children vaccinated with SOBERANA^®^ 02 followed by SOBERANA^®^ Plus compared with children who have recovered from symptomatic COVID-19. The study also provides new evidence on the characterization of the humoral response with respect to the previous findings [17].

Our study demonstrated a higher spike-specific IgG antibody response in vaccinated compared to COVID-19 recovered children, as was found previously for adults vaccinated with the mRNA vaccine [21]. Also, this high level of spike-specific IgG antibodies agrees with published responses in children and adolescents after different vaccination schemes [22,23,24]. Since the humoral response can decay over time in recovered children and adolescents [25,26], the need for a booster immunization in children post-natural infection was suggested [13].

As IgA antibodies predominate in the acute phase of the COVID-19 disease [27], serum RBD-specific IgA antibody levels were determined in vaccinated children and were compared to the COVID-19 recovered group; vaccination induced higher RBD-specific IgA concentrations.

The cellular response differs between vaccinated and recovered children. Previously, we demonstrated that there were not significant changes in hematology analyses post this vaccination [17]. In vaccinated children, there was a higher percentage of switched memory B cells (CD19^+^CD27^+^IgD^−^) compared to convalescent children. No significant difference in naïve, pre-switched, and exhausted B cells between both groups was found. Concerning antibody concentration and total switched memory B cells, there were significant differences between both groups in the RBD^+^ IgG, IgM, and IgA memory subpopulations, with higher values after vaccination. These results significantly correlated with specific antibodies of both isotypes. The association among antibodies, neutralizing capacity, and specific circulating memory B cells have been previously described after infection and vaccination with mRNA vaccines in children [24] and adults [28,29]. Also, it has been reported that the compartment of the memory B cell remains elevated after vaccination with SARS-CoV-2 mRNA vaccines [30,31]. The proportion of circulating memory B cells are also in correspondence with neutralizing antibodies against D614G and VOC, and this phenomenon was described by Cinicola et al., 2022, after vaccination with the BNT162b2 vaccine in children 5–11 y/o [24]. It also has been shown that consecutive exposures to the SARS-CoV-2 spike protein by vaccination increases the neutralizing capacity of antibodies, not only against the Wuhan variant, but also against VOCs [32].

The high level of RBD-specific IgA antibodies and the detection of an IgA memory B cell population suggest protection not only at a systemic level, also in the mucosal compartment by the migration of these cells. This phenomenon has been documented after vaccination with the BNT162b2 vaccine [33]. In addition, the role of the IgA antibodies induced after the Omicron/BA.1 breakthrough infection in mRNA-vaccinated subjects was demonstrated [34].

The generation of an immunological memory is the key for vaccine success. Both memory B and T cells contribute to the maintenance of the immune response over time. In this study, we found that both the RBD-specific antibody response and the relative percentage of the memory B cells increased in vaccinated individuals, suggesting a qualitative and quantitative higher humoral immune response induced by vaccination with SOBERANA^®^ 02/SOBERANA^®^ Plus than after natural infection.

In addition to antibodies, both CD4^+^ T helper (Th) cells and CD8^+^ cytotoxic T lymphocyte (CTL)-specific responses are key factors against viral infections like COVID-19 [35]. Furthermore, each CD4^+^ Th cell subpopulation has different functions and their balance is critical for the control of pathogen infections. We found a higher percentage of Th1 and Th2 CD4^+^ T cells in vaccinated children compared to convalescent individuals. This result is in accordance with previous findings after vaccination with SOBERANA^®^ 02 and SOBERANA^®^ Plus in children in a phase I/II clinical trial, as determined by IFN-γ and IL-4 ELISpot assay [17]. Taken together, these results indicate a mixed post-vaccination response that would have implications not only for the coordination of the T cell-mediated response, especially of CTL, but also for the coordination of the humoral response necessary for viral neutralization. Previous findings indicate that there is a strong correlation between the CD4^+^ T-cell response and the neutralizing antibody response in adults [36,37] and convalescent children [19,38,39]. In correspondence with this work, a decrease in the percentage of CD4^+^ cells with a Th1 phenotype was detected in patients with COVID-19 [40], which has been associated with a bad prognosis and resolution of the disease.

Previous studies showed that the CD8^+^ specific cytotoxic T cell response is critical in the protection against SARS-CoV-2 infection [41]. To evaluate the functionality of these T cell subpopulations, the IFN-γ release in different memory subpopulations was compared after in vitro stimulation with S1 peptides. The analysis showed a predominant effector memory phenotype in individuals vaccinated with the SOBERANA^®^ 02/SOBERANA^®^ Plus scheme, which was comparable to results obtained after vaccination with mRNA-1273 and ChAdOx1 vaccines in children and adolescents [23]. These results are also in accordance with previous data showing that COVID recovered children mostly exhibit a CD4^+^ and CD8^+^ effector memory phenotype [42,43]. Thus, the heterologous scheme presented here is comparable in terms of effector memory capacity to that of natural infection. Additionally, we found high levels of IFN-γ in the supernatant of PBMC, which is in line with the high percentage of activated memory T cells. Dowell et al. also reported that after mRNA-1273 and ChAdOx vaccination, children develop a Th1 response profile [23].

This study has some limitations, including the small number of participants, which does not allow an age-related analysis. Also, we did not study viral neutralization for Omicron variants such as BA.4/BA.5, XBB1.5, and others. Another limitation is that due to the number of cells available, no positive controls were used in the IFN-γ secretion assays. 

## 5. Conclusions 

Our study provides new insights about RBD-specific IgA antibodies, characterizes the phenotype of circulating B and T cells and the specific IFN-γ secretion of CD4^+^ and CD8^+^ T cells after in vitro stimulation with S1 peptides induced by the heterologous SOBERANA^®^ 02/SOBERANA^®^ Plus scheme in children aged 5–11 years, and provides new evidence of the T cells and memory responses in young children. As far as we know, this is the first report of the deep characterization of the cellular immune response with protein vaccines in young children. The heterologous scheme can induce a coordinated humoral and cellular memory response against COVID-19 in the pediatric population, which is qualitatively superior to the response generated by the natural infection. Taken together with its safety profile, the heterologous SOBERANA^®^ 02/SOBERANA^®^ Plus scheme is an excellent alternative for COVID-19 vaccination in children.

## Figures and Tables

**Table 1:** Demographic characteristics of subjects included in the study.

	Vaccinated Children	Children Recovered from COVID-19
N	15	10
Sex		
Female	6 (40.0%)	4 (40.0%)
Male	9 (60.0%)	6 (60.0%)
Skin color		
White	12 (80.0%)	3 (30.0%)
Black	1 (6.6%)	1 (10.0%)
Multiracial	2 (12.3%)	6 (60.0%)
Age (years)		
Mean (SD)	5.3 (2.1)	7.9 (2.9)
Median (IQR)	9.0 (6.0)	7.5 (5.0)
Range	(5; 11)	(4; 11)

## Data Availability

Data are available upon request.

## Supplementary Materials

The following supporting information can be downloaded at: https://www.mdpi.com/article/10.3390/vaccines11111636/s1, Figure S1. Gating strategies for flow cytometry analysis of total B cell subpopulation; Figure S2. Gating strategies for flow cytometry analysis of CD4 T helper subpopulation; Figure S3. Gating strategies for flow cytometry analysis of specific IFN-γ secreting T memory cell in vitro responses; Figure S4. Distribution of B cell subpopulations in vaccinated and COVID-19-recovered children.
