# Supplementary material for: Comparative Immune Response after Vaccination with SOBERANA® 02 and SOBERANA® plus Heterologous Scheme and Natural Infection in Young Children
Source: Vaccines (Basel). 2023 Oct 25;11(11):1636. doi: 10.3390/vaccines11111636 (PMC10675375; doi:10.3390/vaccines11111636)
Supplement: Supplementary file 1 [file vaccines-11-01636-s001.zip › vaccines-2625689-supplementary.pdf]

Supplementary Material

Comparative Immune Response after Vaccination with SOBERANA® 02 and SOBERANA® plus Heterologous Scheme and Natural Infection in Young Children

Rocmira Pérez-Nicado [1], Chiara Massa [2,3], Laura Marta Rodríguez-Noda [1], Anja Müller [3], Rinaldo Puga-Gómez [4], Yariset Ricardo-Delgado [4], Beatriz Paredes-Moreno [1], Meiby Rodríguez-González [1], Marylé García-Ferrer [1], Ilianet Palmero-Álvarez [1], Aniurka Garcés-Hechavarría [1], Daniel G. Rivera [5], Yury Valdés-Balbín [1], Vicente Vérez-Bencomo [1], Dagmar García-Rivera [1,*] and Barbara Seliger [2,3,6,*]

[1] Finlay Vaccine Institute, 200 and 21 Street, Havana 11600, Cuba
[2] Institute for Translational Immunology, Brandenburg Medical School "Theodor Fontane", 14770 Brandenburg, Germany
[3] Medical Faculty, Martin Luther University, 06112 Halle (Saale), Germany
[4] Pediatric Hospital "Juan Manuel Márquez", Havana 11500, Cuba
[5] Laboratory of Synthetic and Biomolecular Chemistry, Faculty of Chemistry, University of Havana, Havana 10400, Cuba
[6] Fraunhofer Institute for Cell Therapy and Immunology, 04103 Leipzig, Germany
* Correspondence: dagarcia@finlay.edu.cu (D.G.-R.); barbara.seliger@uk-halle.de (B.S.)

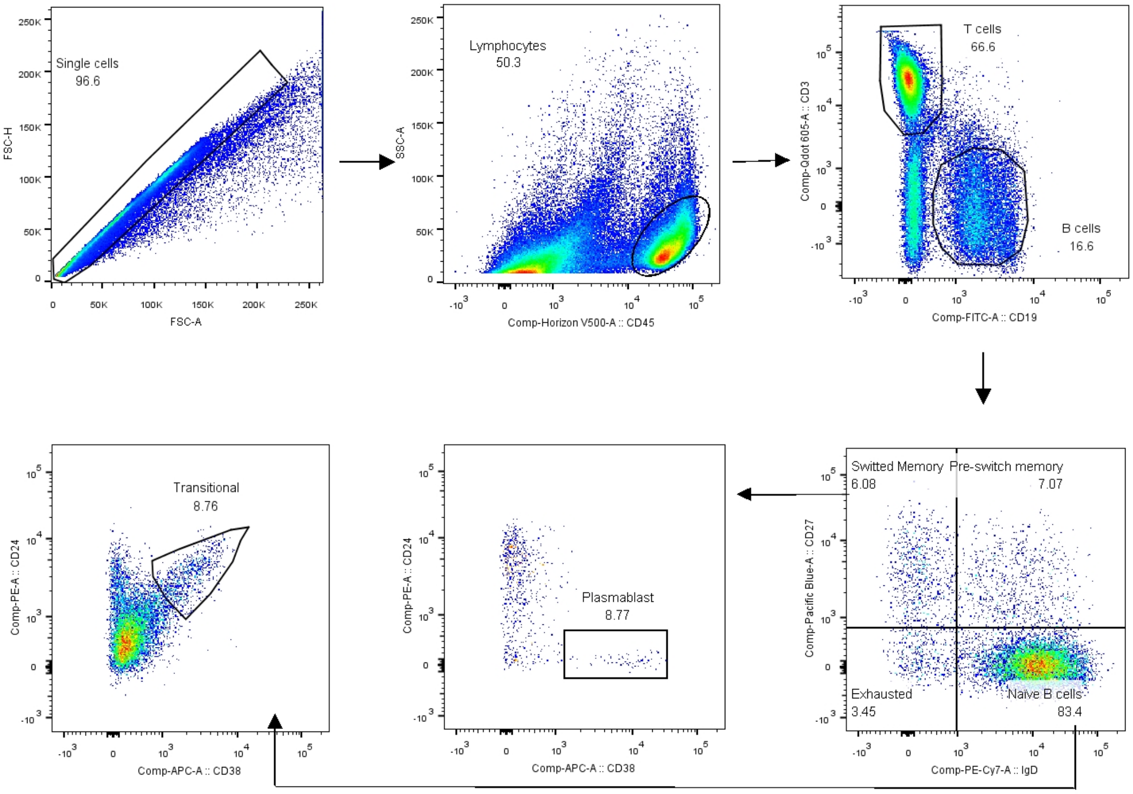

Figure S1. Gating strategies for flow cytometry analysis of total B cell subpopulation. Analysis of total CD19⁺ B cell subpopulations, through surface markers CD27, IgD, CD24 and CD38.

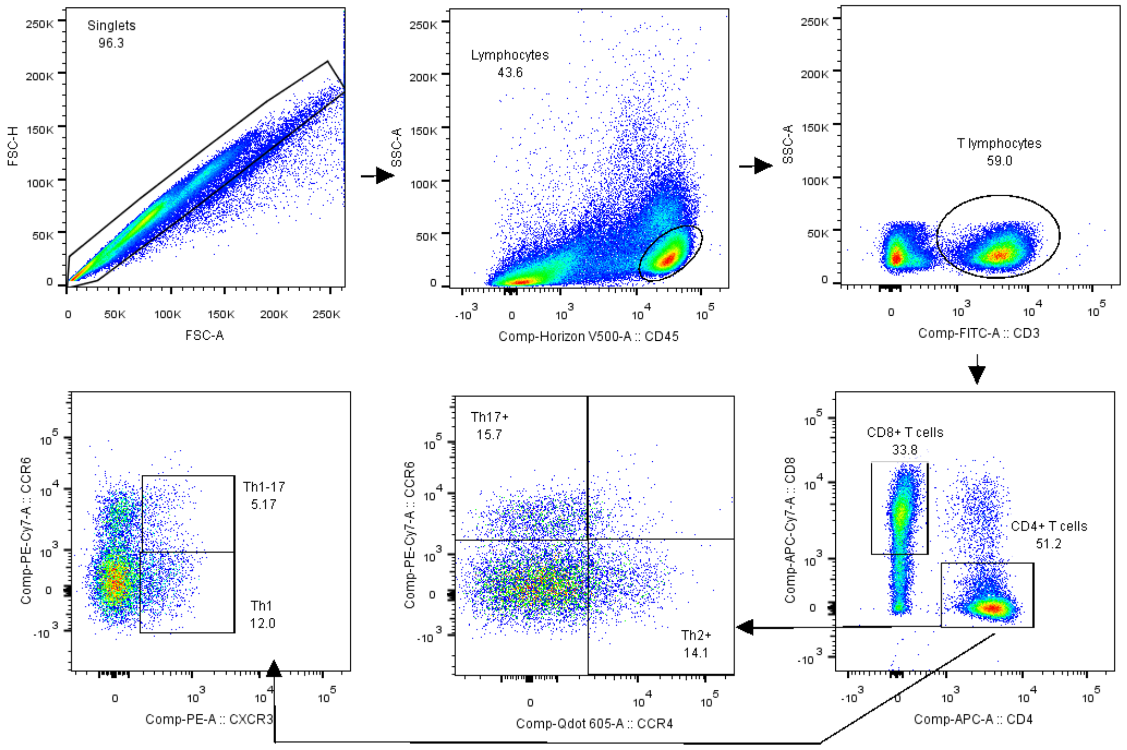

Figure S2. Gating strategies for flow cytometry analysis of CD4 T helper subpopulation. Analysis of T helper subpopulations, through surface markers CCR6, CCR4 and CXCR3.

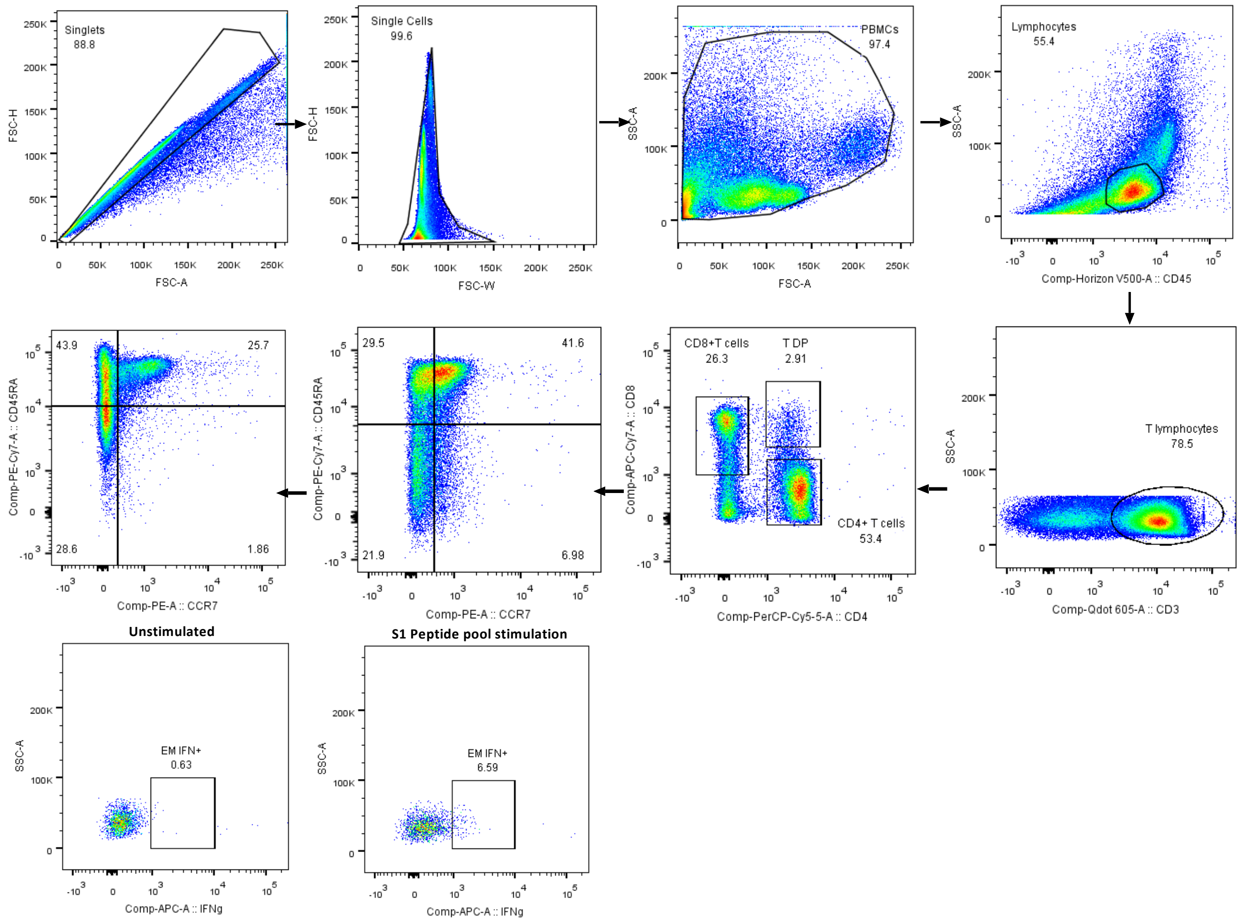

Figure S3. Gating strategies for flow cytometry analysis of specific IFN-γ secreting T memory cell in vitro responses. Analysis of memory CD4 and CD8 cells, through surface markers CCR7, CD45RA and IFN-γ.

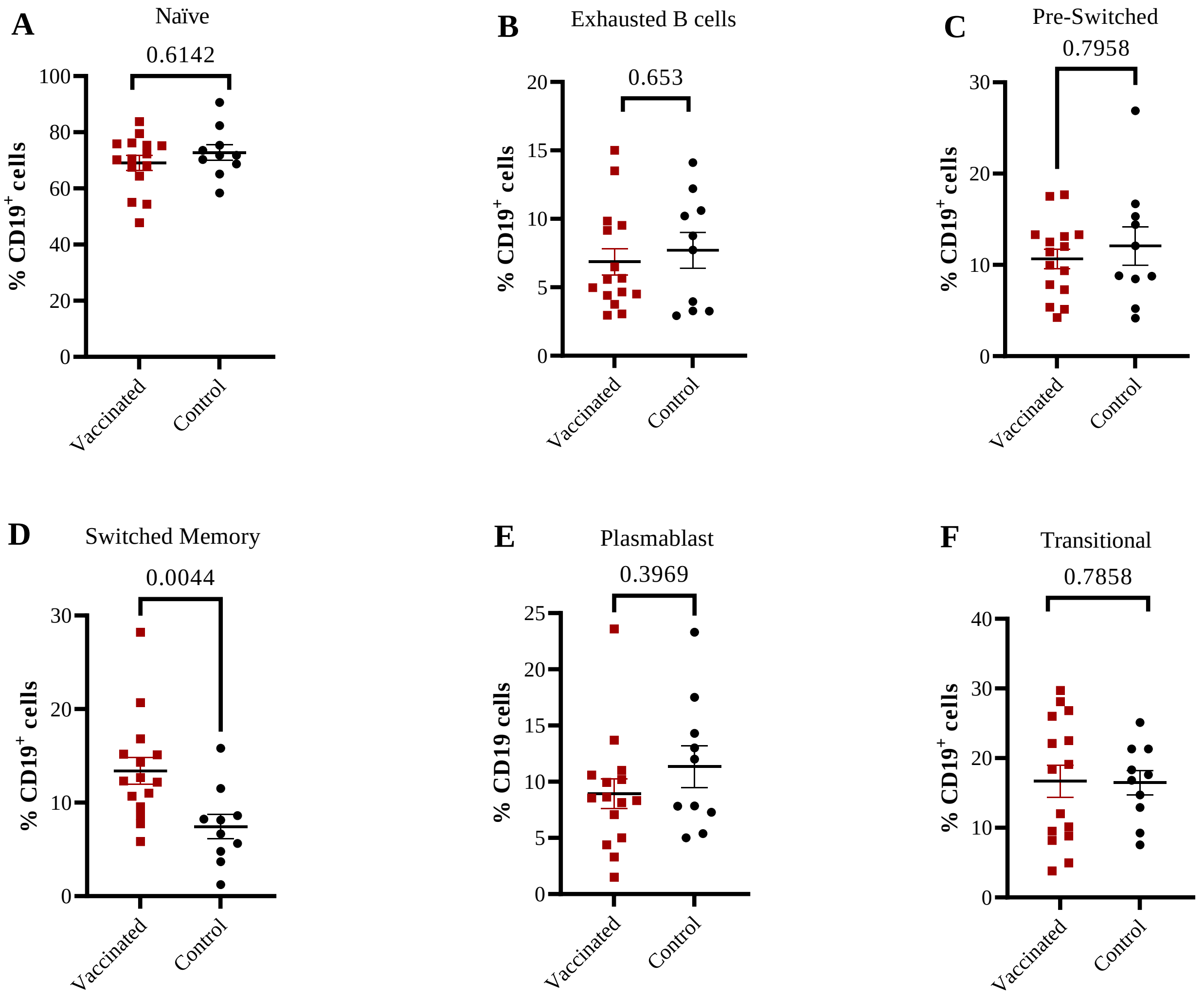

Figure S4: Distribution of B cell subpopulations in vaccinated and COVID-19-recovered children. PBMCs from vaccinated and COVID-19-recovered children (control) were isolated and stained with antibodies cocktails for B cell subpopulations. Frequency of naïve B cells (CD19+IgD+CD27-) (A), exhausted B cells (CD19+IgD-CD27-) (B), pre-switched B cells (CD19+IgD+CD27+) (C), switched B cells (CD19+IgD-CD27+) (D), plasmablast switched B cells (CD19+ IgD-CD27+CD24-CD38+) (E), transitional naïve B cells (CD19+IgD+CD27-CD24+CD38+) (F) are shown for each individual as well as mean ± SEM together with the p value from the Mann-Whitney non-parametric t test.
